# Asset Spend-Down and Medicaid Enrollment in Nursing Homes

**DOI:** 10.1001/jamanetworkopen.2025.46876

**Published:** 2025-12-04

**Authors:** Gabriella Aboulafia, Amanda C. Chen, David C. Grabowski

**Affiliations:** 1 PhD Program in Health Policy, Harvard University, Cambridge, Massachusetts; 2 Department of Pharmaceutical and Health Economics, Mann School of Pharmacy, University of Southern California, Los Angeles; 3 Schaeffer Center for Health Policy & Economics, Los Angeles, California; 4 Department of Health Care Policy, Harvard Medical School, Boston, Massachusetts

## Abstract

**Importance:**

Medicaid eligibility for nursing home care is determined in part by an individual’s (or a couples’, if married) financial resources, including income and assets. To qualify, individuals must “spend down” their resources to meet states’ Medicaid eligibility asset thresholds. Little empirical work has examined the rate of Medicaid spend-down in nursing homes over the past 2 decades.

**Objective:**

To identify the rate of spend-down in nursing homes, defined as the share of total residents who began their stay as non-Medicaid enrolled (after accounting for Medicare-covered skilled nursing facility [SNF] days, where applicable) and became Medicaid enrolled before discharge or death.

**Design, Setting, and Participants:**

This cohort study used a combination of administrative, enrollment, and claims data from 2018 to 2022 to build a panel of 191 416 US nursing home residents enrolled in traditional Medicare—including those admitted for postacute and long-term care—who newly entered a facility in 2018 and either stayed beyond their Medicare SNF days or did not have any Medicare-covered SNF days. Statistical analysis was performed from July 2024 to October 2025.

**Exposure:**

Newly entering a nursing home in 2018 as non-Medicaid enrolled.

**Main Outcomes and Measures:**

The main outcome was whether an individual spent down their assets and became enrolled in Medicaid during their nursing home stay. Multivariate regression was used to identify factors associated with spend-down.

**Results:**

The study included 191 416 individuals (mean [SD] age at time of admission, 81.0 [11.4] years; 58.0% women; mean [SD] time in nursing home, 331.0 [485.8] days) with traditional Medicare who newly entered a nursing home in 2018, of whom 33.9% either began their stay as Medicaid enrolled or enrolled in Medicaid after the completion of their Medicare-covered SNF days. The remaining 66.2% of individuals were initially not enrolled in Medicaid on admission or after the completion of their Medicare-covered SNF days. Of those who were initially not Medicaid enrolled, 16.4% spent down their assets during their stay and enrolled in Medicaid (mean [SD] time to spend-down, 6.1 [7.9] months). The likelihood of spend-down increased with length of stay and was higher among Black, Hispanic, North American Native, and younger residents.

**Conclusions and Relevance:**

In this cohort study of nursing home residents, those who entered a nursing home as initially non-Medicaid enrolled, especially those with longer stays, were at risk of spending down their assets and enrolling in Medicaid. This finding raises concerns both about individuals impoverishing themselves because of the high cost of care and the long-term financial sustainability of the Medicaid program.

## Key Points

**Question:**

What is the rate of Medicaid “spend-down” among nursing home residents enrolled in traditional Medicare?

**Findings:**

In this cohort study of 191 416 US nursing home residents, 16.4% of residents who were initially not enrolled in Medicaid (largely self-pay) who entered a nursing home in 2018 spent down their assets and became enrolled in Medicaid between 2018 and 2022. The likelihood of spending down assets and enrolling in Medicaid varied by demographic characteristics and time spent in a nursing home.

**Meaning:**

This study suggests that Medicaid spend-down has implications for individuals’ and families’ financial well-being and likely the long-term financial sustainability of the Medicaid program.

## Teaser

This cohort study examines the rate of Medicaid spend-down among nursing home residents enrolled in traditional Medicare and assesses what factors are associated with Medicaid spend-down among these residents.

## Introduction

The financing of long-term services and supports (LTSS) is a major policy concern given the aging population and, thus, growing demand for care.^1,2^ Medicaid is the primary LTSS payer, representing over half of all spending.^3^ Medicare generally does not cover LTSS, and only about 11% of older adults have private long-term care insurance.^2,3^ Older adults and those with disabilities—the primary LTSS users—make up less than one-fourth of the Medicaid population but account for over half of all Medicaid spending.^4^

Eligibility for Medicaid for older adults and those with disabilities depends partly on income and assets. If an individual is married, the couples’ resources are used in the eligibility determination (although spousal impoverishment rules for nursing home care protect some resources for community-living spouses^5^). To qualify, individuals must “spend down” their resources to meet states’ Medicaid eligibility thresholds. The annual cost of LTSS—which can range from $24 700 to $288 288 depending on the service—far exceeds the median income among older adults ($36 000) and easily could surpass the entirety of the median older adult’s savings ($103 800),^6^ raising concerns about people impoverishing themselves to qualify for the program.

The COVID-19 pandemic reignited the decades-old policy conversation about the broader issue of LTSS financing, at least temporarily.^7^ However, there has been little empirical work or policy discussion on the spend-down rate in nursing homes over the past 2 decades (there has, however, been more recent work on Medicaid spend-down for LTSS services more broadly, suggesting a spend-down rate of roughly 10%^8,9^).

The use of home and community-based services has increased over the past several decades, but nursing home care was historically the primary form of LTSS,^10^ leading to more focus on spend-down in institutional settings.^11^ Most studies of nursing home spend-down were published in the 1980s and early 1990s. Moreover, each study uses a different spend-down measure, leading to highly variable estimates.^12^ Studies that provide the most complete picture of spend-down in nursing homes—defined as the share of total residents who were not enrolled in Medicaid at admission and enrolled in Medicaid on discharge—estimate spend-down to be between 10%^13^ and 27%.^14^

The objective of this study is to identify how many nursing home residents transitioned from non-Medicaid–enrolled to Medicaid-enrolled status during their nursing home stay (and thus “spent down” their financial resources). We also examine factors associated with spend-down over time. Our study adds to the literature by providing an updated examination of Medicaid spend-down among nursing home residents.

## Methods

This cohort study was approved by the Harvard Medical School institutional review committee, which waived the informed consent requirement because the study used deidentified data. We followed the Strengthening the Reporting of Observational Studies in Epidemiology (STROBE) reporting guideline for cohort studies.

### Data Sources and Sample Construction

To assess the rate of Medicaid spend-down among nursing home residents, we constructed a cohort of traditional Medicare beneficiaries who newly entered a nursing home in 2018 and followed them for up to 5 years (through 2022) using the following data sources:

1. The Minimum Data Set (MDS) 3.0, 2018-2022,^15^ to identify and follow up with nursing home residents throughout their stay.
2. The Master Beneficiary Summary File (MBSF), 2018-2022,^16^ to identify monthly Medicaid eligibility status for our cohort of nursing home residents.
3. The Medicare Provider Analysis and Review (MedPAR) file, 2018-2019,^17^ to identify whether a nursing home stay was initially covered by Medicare, and the length of that coverage.

Our cohort of nursing home residents consisted of 191 416 individuals, including those admitted for postacute and long-term care (eFigure 1 in Supplement 1). To obtain our final cohort of residents, we first identified those who were newly admitted to a nursing home in the 2018 MDS, excluding those with a nursing home stay in the prior year. This ensured that we captured individuals at the start of a stay before any nursing home–related spending occurred. We tracked our cohort’s use of nursing homes through 2022. Given our interest in how time spent in a nursing home is associated with transitions to Medicaid eligibility, we limited our sample to residents with clean stays, defined as those with no gaps in their stay, or gaps of 30 days or less between stays.

Then, we linked our MDS cohort to the MBSF to obtain monthly Medicaid enrollment status. We limited our cohort to Medicare beneficiaries as of January 2018. We also excluded individuals who were partially dually enrolled in Medicare and Medicaid at any point of the study period.

Finally, we merged our MDS-MBSF sample with MedPAR claims to identify Medicare-covered nursing home days. Traditional Medicare generally covers up to 100 days of care in a skilled nursing facility (SNF) after a qualifying hospitalization. Because the MedPAR file reliably includes claims only for Medicare-covered SNF days for traditional Medicare enrollees,^18^ our analysis is limited to those with traditional Medicare. This choice is consistent with prior work examining Medicare coverage of SNF stays.^19^ The claims allow us to observe whether and for how long Medicare covered a resident’s stay. A small share of individuals covered by traditional Medicare may face some cost sharing starting on day 21 of their SNF stay if they do not qualify for Medicaid or have supplemental coverage (eg, Medigap).^20^ Additional details on how we matched Medicare-covered SNF days in the MedPAR with nursing home stays in the MDS are included in the eMethods in Supplement 1.

We limited our sample to those who remained in a facility beyond the Medicare-covered portion of their stay (ie, excluding those with only Medicare-covered SNF days) and those who did not have any Medicare-covered SNF days. We made this choice because we are interested in the association between time spent in a nursing home and transitions to Medicaid, and individuals with entirely Medicare-covered SNF stays are never observed starting with or transitioning to Medicaid.

After residents transitioned out of their Medicare-covered SNF days (if applicable), we did not account for any subsequent Medicare-covered SNF days that may have occurred later because of hospitalization. These days do not contribute meaningfully to spend-down, with the exception of some cost sharing.

### Key Variables

#### Time

When referencing time spent in a nursing home (ie, length of stay), we defined day 1 as the first day an individual remained in a nursing home beyond their Medicare-covered SNF days, if applicable. For individuals admitted directly from the community without any Medicare-covered SNF days, day 1 was the true first day of their nursing home stay. If an individual was discharged from a nursing home (with a gap between stays of ≤30 days, as mentioned), these days were not counted toward the time spent during a stay; the count resumed if they returned to a facility. We identified the final day of a resident’s stay based on their date of death indicated by the MBSF, or discharge date or last date indicated by the MDS.

#### Medicaid Enrollment and Spend-Down

We measured spend-down as a change in Medicaid enrollment over an individual’s nursing home stay. We created a binary indicator equal to 1 if an individual was Medicaid enrolled and 0 if the individual was non-Medicaid enrolled in each month, using the MBSF’s monthly Medicaid enrollment information. Most nursing home residents who are not Medicaid enrolled pay for their care out of pocket because few individuals have private long-term care insurance or other public insurance (eg, through the Veterans Health Administration).

We defined spend-down in nursing homes as a transition from non-Medicaid enrolled to Medicaid enrolled. When we reference those who were initially non-Medicaid enrolled, we are referring to individuals who entered their stay as non-Medicaid enrolled or were immediately non-Medicaid enrolled after their Medicare-covered SNF days. When we reference those who were initially Medicaid enrolled, we are referring to individuals who entered their stay as Medicaid enrolled or were immediately Medicaid enrolled after their Medicare-covered SNF days.

### Statistical Analysis

#### Main Analyses

Statistical analysis was performed from July 2024 to October 2025 using Stata, version 18.0 (StataCorp LLC). We first examined the share of our sample that was not initially Medicaid enrolled among the full sample, and then we examined the sample stratified by time spent in a nursing home (eg, 3 and 6 months and 1, 2, 3, and 4 years). At each time point, the numerator was the number of living individuals in a nursing home who were initially Medicaid enrolled, and the denominator was all living individuals in a nursing home at that same time. When we stratified our sample by these time points, the sample size changed (ie, decreased as time spent in a nursing home increased). The sample size changed both due to residents being discharged and death. We include information about the number of individuals who were discharged or died before each time point in eTables 1 and 2 in Supplement 1.

We then examined the spend-down rate among individuals initially non-Medicaid enrolled, again first among the full sample and then stratified by time spent in a nursing home. At each time point, the numerator was the number of living individuals in a nursing home who had spent down by the given time point, and the denominator was all living individuals in a nursing home who were initially non-Medicaid enrolled at that same time.

Using multivariate linear probability models, we then assessed the demographic characteristics associated with the likelihood of spend-down among those initially non-Medicaid enrolled. We ran a model for each time point to examine whether there was change in the characteristics associated with the spend-down based on length of stay. Our models included race and ethnicity (Asian, Black, Hispanic, North American Native, White, and other race and ethnicity [which includes individuals of unspecified other race or ethnicity and those with unknown race or ethnicity]) (from the MBSF); sex, age, and state of residence (from the MBSF); and marital status and length of nursing home stay (from the MDS). Data on race and ethnicity were collected because race and ethnicity are important demographic characteristics commonly controlled for in studies of nursing home use. Statistical significance was assessed at 2-sided *P* < .05. Predicted probabilities were estimated using Stata’s postestimation margins command.

#### Supplemental Analyses

We conducted 2 supplementary analyses. First, we replicated our main analysis using a 2015-entry cohort followed through 2019, to assess the robustness of our findings. Second, given concerns that the COVID-19 pandemic might have led to compositional shifts in the nursing home population,^21^ we assessed the spend-down rate just before and during the pandemic, using 2018- and 2020-entry cohorts, each followed for 2 years. We include this analysis both to ensure the inclusion of the pandemic data years in our main 2018 cohort did not bias our results and to examine whether the spend-down rate in nursing homes differed during the pandemic.

## Results

### Study Sample and Descriptive Statistics

Our sample comprised 191 416 nursing home residents (mean [SD] age at time of admission, 81.0 [11.4] years; 111 053 women [58.0%] and 80 363 men [42.0%]; 3682 Asian residents [1.9%], 16 089 Black residents [8.4%], 3087 Hispanic residents [1.6%], 1145 North American Native residents [0.6%], 163 780 White residents [85.6%], and 3633 residents of other race or ethnicity [1.9%]) who newly entered a nursing home in 2018 and were covered by traditional Medicare; 64 790 (33.9%) entered their stay initially Medicaid enrolled (because they either began their stay as Medicaid enrolled or were Medicaid enrolled after their Medicare-covered SNF days concluded), and 126 626 (66.2%) were initially non-Medicaid enrolled (because they either began their stay as non-Medicaid enrolled or were non-Medicaid enrolled after their Medicare-covered SNF days concluded) (Table 1). The mean (SD) length of time spent in a nursing home was 331.0 (485.8) days (about 11 months).

**Table 1.** Characteristics of Study Sample^a^

Characteristic	Nursing home residents, No. (%)^b^		
	Overall (N = 191 416)	Initially Medicaid enrolled (n = 64 790 [33.9%])	Initially non-Medicaid enrolled (n = 126 626 [66.2%])
Age, mean (SD), y	81.0 (11.4)	78.0 (12.8)	82.5 (10.4)
Sex			
Female	111 053 (58.0)	40 581 (62.6)	70 472 (55.7)
Male	80 363 (42.0)	24 209 (37.4)	56 154 (44.4)
Marital status			
Married	61 206 (32.0)	12 676 (19.6)	48 530 (38.3)
Widowed, separated, or divorced	98 567 (51.5)	34 907 (53.9)	63 660 (50.3)
Never married	24 534 (12.8)	14 595 (22.5)	9939 (7.9)
Unknown marital status	7109 (3.7)	2612 (4.0)	4497 (3.6)
Race and ethnicity			
Asian	3682 (1.9)	2743 (4.2)	939 (0.7)
Black	16 089 (8.4)	9244 (14.3)	6845 (5.4)
Hispanic	3087 (1.6)	2437 (3.8)	650 (0.5)
North American Native	1145 (0.6)	686 (1.1)	459 (0.4)
White	163 780 (85.6)	48 082 (74.2)	115 698 (91.4)
Other^c^	3633 (1.9)	1598 (2.5)	2035 (1.6)
Entered nursing home with Medicare-covered SNF days			
Yes	90 077 (47.1)	34 913 (53.9)	55 164 (43.6)
No	101 339 (52.9)	29 877 (46.1)	71 462 (56.4)
Length of nursing home stay, mean (SD), d^d^	331.0 (485.8)	496.0 (553.9)	246.6 (422.7)

Abbreviation: SNF, skilled nursing facility.

^a^ Data are from the authors’ analysis of the Minimum Dataset, 2018-2022; Master Beneficiary Summary File, 2018-2022; and Medicare Provider Analysis and Review file, 2018-2019.

^b^ Initially Medicaid enrolled individuals include those who were Medicaid enrolled on admission or after the end of the Medicare-covered part of their stay, if applicable. Initially non-Medicaid enrolled individuals include those who were non-Medicaid enrolled on admission or after the end of the Medicare-covered part of their stay, if applicable.

^c^ Includes individuals of unspecified other race or ethnicity and those with unknown race or ethnicity.

^d^ For individuals who entered a nursing home with Medicare-covered SNF days, the number of days an individual remained in a nursing home beyond their Medicare-covered SNF days is referenced. For individuals admitted directly from the community without any Medicare-covered SNF days, the true amount of time spent in a nursing home is referenced.

A total of 90 077 individuals (47.1%) entered a nursing home with Medicare-covered SNF days covering the first part of their stay (Table 1). This estimate is substantially lower than reported in prior research,^19^ mostly reflecting a mechanical feature of our sample construction, as our sample excludes those whose nursing home stay was entirely covered under the Medicare SNF benefit (described in the Methods).

### Nursing Home Residents Initially Medicaid Enrolled, by Length of Stay

**Figure 1.**
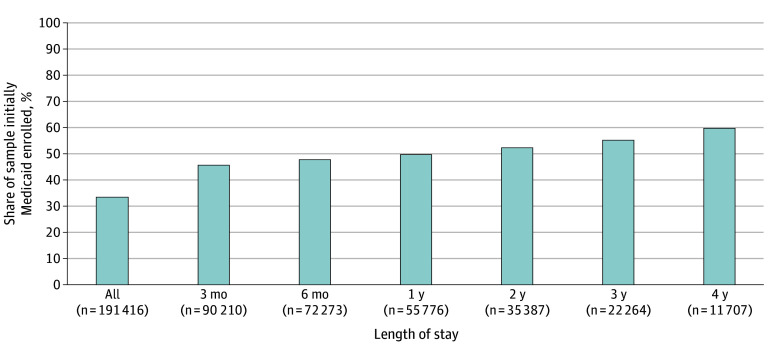
Share of Nursing Home Residents Who Were Initially Medicaid Enrolled, by Length of Nursing Home Stay The graph shows the share of 2018 new nursing home entrants who were initially Medicaid enrolled: those Medicaid enrolled on admission or after the end of their Medicare-covered skilled nursing facility days, if applicable. In the “All” column, the denominator is the full sample, not conditioned by any time point. “Length of nursing home stay” references the number of days an individual remained in a nursing home beyond their Medicare-covered skilled nursing facility days, if applicable. For individuals admitted directly from the community without any Medicare-covered skilled nursing facility days, “Length of nursing home stay” references the true amount of time spent in a nursing home. Data are from the authors’ analysis of the Minimum Dataset, 2018-2022; Master Beneficiary Summary File, 2018-2022; and Medicare Provider Analysis and Review file, 2018-2019.

The share of those initially Medicaid enrolled (ie, individuals who began their stay as Medicaid enrolled or were Medicaid enrolled after their Medicare-covered SNF days concluded) remained sizable over time (Figure 1). As also noted in Table 1, 33.9% of the full sample (n = 64 790) was Medicaid enrolled at admission or by completion of their Medicare-covered SNF days. The share of nursing home residents who were initially Medicaid enrolled increased as the length of stay increased. By 4 years, the share of residents who were initially Medicaid enrolled comprised 60.3% (7063 of 11 707) of all living nursing home residents in our sample.

### Spend-Down Among Nursing Home Residents Initially Non-Medicaid Enrolled, by Length of Stay

**Figure 2.**
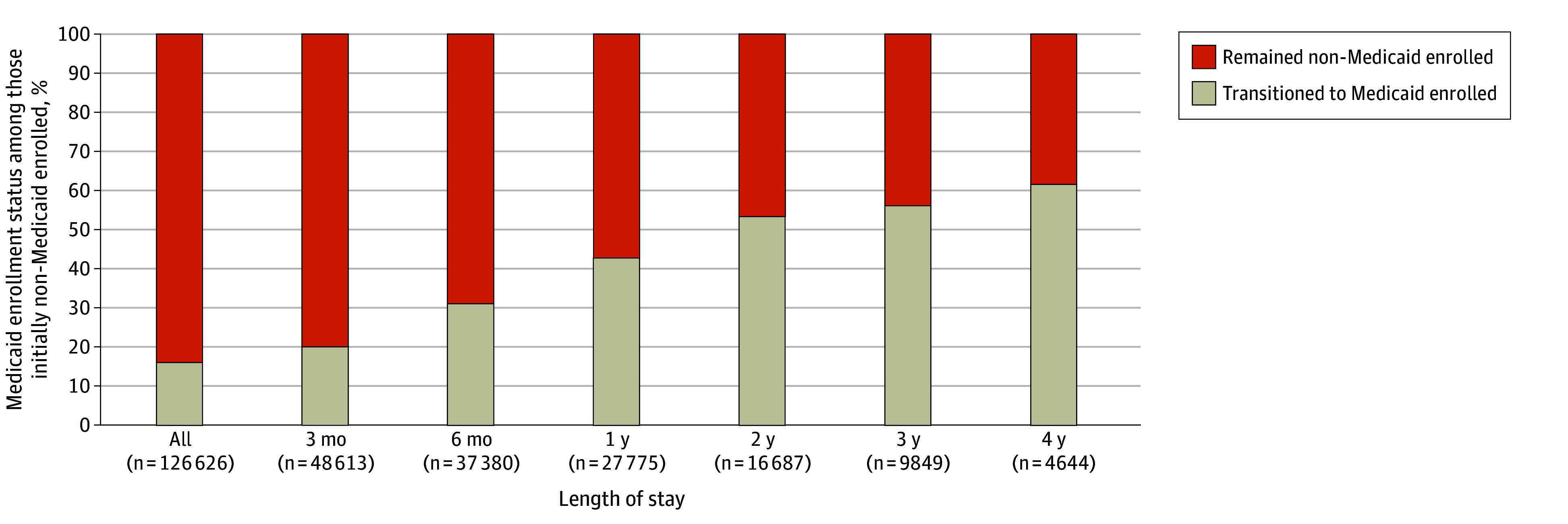
Spend-Down Rate Among Nursing Home Residents Not Initially Medicaid Enrolled, by Length of Nursing Home Stay The graph shows Medicaid enrollment status among those initially non-Medicaid enrolled: those non-Medicaid enrolled on admission or after the end of their Medicare-covered skilled nursing facility days, if applicable. In the “All” column, the denominator is the full sample who were initially non-Medicaid enrolled, not conditioned by any time point. “Length of nursing home stay” references the number of days an individual remained in a nursing home beyond their Medicare-covered skilled nursing facility days, if applicable. For individuals admitted directly from the community without any Medicare-covered skilled nursing facility days, “Length of nursing home stay” references the true amount of time spent in a nursing home. Data are from the authors’ analysis of the Minimum Dataset, 2018-2022; Master Beneficiary Summary File, 2018-2022; and Medicare Provider Analysis and Review file, 2018-2019.

Of those who were initially non-Medicaid enrolled, 16.4% (20 773 of 126 626) spent down their assets and became Medicaid enrolled (Figure 2). However, the likelihood of spend-down increased as time spent in a nursing home increased. By 3 months, 20.5% of living initially non-Medicaid enrolled individuals (9941 of 48 613) had spent down; by 4 years, 61.8% (2871 of 4644) had done so. Among the initially non-Medicaid–enrolled group, the mean (SD) time until spend-down was approximately 6.1 (7.9) months (eFigure 2 in Supplement 1). We found these results to be very similar when using a 2015-entry cohort, as well as before and during the COVID-19 pandemic (eFigures 3 and 4 in Supplement 1).

### Likelihood of Spend-Down by Demographic Characteristics and Length of Stay

Table 2 shows the likelihood of spend-down among those initially non-Medicaid enrolled by demographic characteristics and time in a nursing home. There were notable differences between demographic subgroups, most of which increased over time. At each time point, Black individuals were between 10 and 17 percentage points more likely to spend down than White individuals. Hispanic and North American Native individuals were between 8 and 15 percentage points more likely to spend down than White individuals among those alive and in a nursing home at 3 and 6 months and 1 and 2 years. The likelihood of spend-down decreased with age—a trend that was consistent at each time point. Those aged 65 years or older were between 4 and 27 percentage points less likely to spend down than those younger than 65 years. Women and individuals who were widowed, separated, or divorced on admission were 3 to 14 percentage points more likely to spend down compared with men and those who were married, respectively. Last, each additional day in a nursing home was associated with an increased likelihood of spend-down at most time points.

**Table 2.** Likelihood of Spend-Down Among Individuals Initially Non-Medicaid Enrolled by Demographic Characteristics and Length of Nursing Home Stay^a^

Characteristic	At 3 mo (n = 48 613)		At 6 mo (n = 37 380)		At 1 y (n = 27 775)		At 2 y (n = 16 687)		At 3 y (n = 9849)		At 4 y (n = 4644)	
	Predicted probability, %	Difference (95% CI)	Predicted probability, %	Difference (95% CI)	Predicted probability, %	Difference (95% CI)	Predicted probability, %	Difference (95% CI)	Predicted probability, %	Difference (95% CI)	Predicted probability, %	Difference (95% CI)
Race and ethnicity												
Asian	18.2	−0.02 (−0.07 to 0.03)	29.3	−0.01 (−0.08 to 0.05)	33.3	−0.09 (−0.18 to −0.004)^b^	46.4	−0.06 (−0.18 to 0.05)	48.4	−0.07 (−0.22 to 0.07)	82.7	0.21 (−0.001 to 0.43)
Black	29.5	0.10 (0.08 to 0.11)^c^	44.1	0.13 (0.11 to 0.16)^c^	59.7	0.17 (0.15 to 0.20)^c^	70.2	0.17 (0.14 to 0.21)^c^	70.5	0.15 (0.10 to 0.19)^c^	72.3	0.11 (0.05 to 0.17)^b^
Hispanic	29.7	0.10 (0.04 to 0.15)^b^	40.0	0.09 (0.01 to 0.17)^b^	57.6	0.15 (0.06 to 0.25)^b^	66.1	0.13 (0.01 to 0.25)^b^	68.7	0.13 (−0.004 to 0.27)	72.5	0.11 (−0.08 to 0.30)
North American Native	27.8	0.08 (0.02 to 0.14)^b^	44.2	0.13 (0.06 to 0.21)^c^	57.2	0.15 (0.06 to 0.24)^b^	65.2	0.12 (0.01 to 0.24)^b^	68.4	0.13 (−0.01 to 0.27)	64.9	0.04 (−0.13 to 0.21)
White	20.0	[Reference]	30.7	[Reference]	42.3	[Reference]	52.9	[Reference]	55.7	[Reference]	61.2	[Reference]
Other^d^	19.1	−0.01 (−0.04 to 0.02)	29.1	−0.02 (−0.06 to 0.03)	37.9	−0.04 (−0.10 to 0.01)	49.1	−0.04 (−0.11 to 0.04)	48.1	−0.08 (−0.17 to 0.02)	54.4	−0.07 (−0.19 to 0.05)
Age groups, y												
<65	27.8	[Reference]	45.4	[Reference]	63.9	[Reference]	75.3	[Reference]	76.4	[Reference]	80.3	[Reference]
65-74	23.9	−0.04 (−0.06 to −0.01)^b^	39.0	−0.06 (−0.10 to −0.03)^c^	53.1	−0.11 (−0.15 to −0.07)^c^	62.9	−0.12 (−0.17 to −0.07)^c^	65.7	−0.11 (−0.17 to −0.05)^c^	65.8	−0.15 (−0.22 to −0.07)^c^
75-84	23.3	−0.05 (−0.07 to −0.02)^c^	35.2	−0.10 (−0.13 to −0.07)^c^	47.8	−0.16 (−0.20 to −0.12)^c^	58.8	−0.16 (−0.21 to −0.11)^c^	60.6	−0.16 (−0.21 to −0.10)^c^	65.8	−0.14 (−0.22 to −0.07)^c^
≥85	18.1	−0.10 (−0.12 to −0.07)^c^	27.7	−0.18 (−0.21 to −0.14)^c^	38.2	−0.26 (−0.30 to −0.22)^c^	47.9	−0.27 (−0.32 to −0.22)^c^	49.7	−0.27 (−0.32 to −0.21)^c^	56.1	−0.24 (−0.31 to −0.17)^c^
Sex												
Female	21.5	0.03 (0.02 to 0.03)^c^	33.0	0.04 (0.03 to 0.05)^c^	45.4	0.07 (0.06 to 0.08)^c^	56.5	0.09 (0.08 to 0.11)^c^	58.9	0.09 (0.07 to 0.11)^c^	65.8	0.14 (0.11 to 0.17)^c^
Male	18.8	[Reference]	28.6	[Reference]	38.6	[Reference]	47.2	[Reference]	49.7	[Reference]	51.6	[Reference]
Marital status												
Married	18.8	[Reference]	29.0	[Reference]	40.4	[Reference]	48.3	[Reference]	50.4	[Reference]	55.5	[Reference]
Never married	19.4	0.01 (−0.01 to 0.02)	30.2	0.01 (−0.01 to 0.03)	42.6	0.02 (−0.001 to 0.04)	51.5	0.03 (0.003 to 0.06)^b^	54.8	0.04 (0.01 to 0.08)^b^	64.1	0.09 (0.04 to 0.14)^b^
Widowed, separated, or divorced	21.4	0.03 (0.02 to 0.03)^c^	32.5	0.03 (0.02 to 0.05)^c^	44.1	0.04 (0.02 to 0.05)^c^	55.7	0.07 (0.06 to 0.09)^c^	58.6	0.08 (0.06 to 0.11)^c^	63.5	0.08 (0.04 to 0.11)^c^
Unknown marital status	21.2	0.02 (0.0001 to 0.05)^b^	34.5	0.05 (0.02 to 0.09)^b^	46.4	0.06 (0.02 to 0.10)^b^	61.0	0.13 (0.07 to 0.18)^c^	62.4	0.12 (0.05 to 0.19)^b^	67.6	0.12 (0.01 to 0.23)^b^
Length of nursing home stay (≥1 d)^e^	NA	0.00009 (0.00008 to 0.0001)^b^	NA	0.00008 (0.00007 to 0.00009)^c^	NA	0.00004 (0.00003 to 0.00005)^c^	NA	0.00001 (−0.00001 to 0.00003)	NA	−0.00006 (−0.0001 to −0.00001)^b^	NA	0.000007 (−0.0001 to 0.0002)

Abbreviations: NA, not applicable; SNF, skilled nursing facility.

^a^ Results from adjusted linear probability models examining characteristics associated with spend-down by each time point among those who were initially non-Medicaid enrolled, alive, and in a nursing home at the same time point. Predicted probabilities were estimated using Stata’s postestimation margins command. In addition to the covariates shown, each model includes state fixed effects. Data are from the authors’ analysis of the Minimum Dataset, 2018-2022; Master Beneficiary Summary File, 2018-2022; and Medicare Provider Analysis and Review file, 2018-2019.

^b^ Indicates statistical significance at *P* < .05.

^c^ Indicates statistical significance at *P* < .001.

^d^ Includes individuals of unspecified other race or ethnicity and those with unknown race or ethnicity.

^e^ For individuals who entered a nursing home with Medicare-covered SNF days, the number of days an individual remained in a nursing home beyond their Medicare-covered SNF days is referenced. For individuals admitted directly from the community without any Medicare-covered SNF days, the true amount of time spent in a nursing home is referenced.

## Discussion

In this cohort study of nursing home residents—all of whom were traditional Medicare beneficiaries and newly entered a nursing home in 2018—we found that about one-third of individuals began their stay initially Medicaid enrolled, as they were Medicaid enrolled on admission or after the completion of their Medicare-covered SNF days. The share of our sample who were initially Medicaid enrolled and remained in a nursing home remained sizable over time. Among those who were initially non-Medicaid enrolled, because they were not Medicaid enrolled on admission or after their Medicare-covered SNF days, 16.4% of individuals spent down and transitioned to Medicaid enrollment.

We show that the risk of spend-down was not distributed equally across our sample, with those from racial and ethnic minority groups—including Black, Hispanic, and North American Native individuals—being significantly more likely to spend down than White individuals. These differences likely reflect preexisting racial and ethnic disparities in wealth.^22^ Older adults were also less likely than younger individuals to spend down, likely reflecting increased savings among older adults. Finally, we found that the likelihood of spend-down and the time in a nursing home were positively associated, as individuals had more time to spend down their resources on nursing home care.

Although our study provides an updated look at the spend-down rate in nursing homes, it underscores the need for more accurate measures of the broader spend-down phenomenon. Spend-down in the community prior to nursing home admission has always been prevalent, but the rapid emergence of privately financed assisted living and other community options over the past 3 decades may have had profound implications for the spend-down rate.^23,24^ Non-Medicaid–enrolled individuals who once spent their savings on nursing home care now may deplete their assets in the assisted living sector and then transition to a nursing home at or near Medicaid eligibility.^9^

Our study also suggests that the spend-down process could have significant financial implications for families. Although we do not evaluate whether families may be shielding their assets from the spend-down process through wealth transfers, prior research has found that asset transfers are relatively rare and small.^25,26,27,28,29^ Last, while we did not examine the association of spend-down with overall Medicaid enrollment and spending, spend-down likely increased both by shifting more people from self-pay to Medicaid, which may exacerbate the program’s LTSS-related financial challenges.^30^

### Limitations

Our study has several limitations. First, the MBSF distinguishes between Medicaid and non-Medicaid payer status only, preventing the identification of individuals with private long-term care insurance, which protects against the risk of spend-down. Second, our ability to fully capture and assess Medicare-covered SNF days was limited. Individuals who are not dually eligible and lack any Medicare supplemental coverage (eg, Medigap) may face some out-of-pocket costs beginning on day 21 of their Medicare-covered SNF days. Although we cannot identify which individuals may have faced some cost sharing, research suggests most Medicare beneficiaries face low exposure to SNF cost sharing due to supplemental coverage.^20^ Third, states vary in their Medicaid eligibility rules. We control for cross-state differences in spend-down in our regression analyses, but we do not explicitly assess how different eligibility rules influence spend-down. Fourth, although we observe length of time until spend-down, we cannot observe how much and on what individuals are spending down. Similarly, while we observe Medicaid enrollment and transitions to Medicaid enrollment using the MBSF, we cannot observe Medicaid eligibility or transitions to Medicaid eligibility (which may have also occurred among our study sample).

Fifth, our study has several limitations in terms of generalizability. We document spend-down conditional on being in a nursing home, missing what happens to individuals before they enter a nursing home, which could be associated with the rate at which they exhaust their assets (including by spending on other kinds of LTSS) on admission to a facility. In addition, our results are specific to those who newly entered a nursing home in 2018. Although we found similar results to our main analysis using a 2015-entry cohort, subsequent cohorts may experience different trends. Our analysis is also restricted to those with traditional Medicare, limiting our ability to generalize to the full Medicare-covered population, including those with Medicare Advantage. Last, the spend-down rates we document by time points (Figure 2) are not directly comparable because the sample at each time point is different.

## Conclusions

In this cohort study of nursing home residents with traditional Medicare, we found that Medicaid spend-down may pose a significant financial risk for nursing home residents, especially those with longer stays. Concerns surrounding spend-down—and the financing of LTSS more generally—are not new. Our study points to the need for a deeper understanding of spend-down, as well as consideration of policy options to both alleviate the financial strain facing families in need of nursing home care and ensure the long-term sustainability of the Medicaid program.

## Notes

Supplement 1.
eMethods.
**eFigure 1.** CONSORT Diagram of Nursing Home Resident Cohort Data Merging Process **eTable 1.** Total Number of Nursing Home Residents Who Were Alive and in a Nursing Home by Given Time Points, Full Sample **eTable 2.** Total Number of Nursing Home Residents Who Were Alive and in a Nursing Home by Given Time Points, Limited to Those Initially Non-Medicaid-Enrolled **eFigure 2.** Time Until Spend-Down Among Nursing Home Residents Initially Non-Medicaid-Enrolled **eFigure 3.** Spend-Down Rate Among Nursing Home Residents Initially Non-Medicaid-Enrolled (2015-2019 Cohort), by Length of Stay **eFigure 4.** Spend-Down Rate Among Nursing Home Residents Initially Non-Medicaid-Enrolled Before (2018-2019) and During (2020-2021) the Pandemic, by Length of Stay

Supplement 2.
Data Sharing Statement
