# Supplementary material for: Asset Spend-Down and Medicaid Enrollment in Nursing Homes
Source: JAMA Netw Open. 2025 Dec 4;8(12):e2546876. doi: 10.1001/jamanetworkopen.2025.46876 (PMC12679321; doi:10.1001/jamanetworkopen.2025.46876)
Supplement: Supplement 1. — eMethods. eFigure 1. CONSORT Diagram of Nursing Home Resident Cohort Data Merging Process eTable 1. Total Number of Nursing Home Residents Who Were Alive and in a Nursing Home by Given Time Points, Full Sample eTable 2. Total Number of Nursing Home Residents Who Were Alive and in a Nursing Home by Given Time Points, Limited to Those Initially Non-Medicaid-Enrolled eFigure 2. Time Until Spend-Down Among Nursing Home Residents Initially Non-Medicaid-Enrolled eFigure 3. Spend-Down Rate Among Nursing Home Residents Initially Non-Medicaid-Enrolled (2015-2019 Cohort), by Length of Stay eFigure 4. Spend-Down Rate Among Nursing Home Residents Initially Non-Medicaid-Enrolled Before (2018-2019) and During (2020-2021) the Pandemic, by Length of Stay [file jamanetwopen-e2546876-s001.pdf]

Supplementary Online Content

Aboulafia G, Chen AC, Grabowski DC. Asset spend-down and Medicaid enrollment in nursing homes. *JAMA Netw Open.* 2025;8(12):e2546876.
doi:10.1001/jamanetworkopen.2025.46876

eMethods.
eFigure 1. CONSORT Diagram of Nursing Home Resident Cohort Data Merging Process
eTable 1. Total Number of Nursing Home Residents Who Were Alive and in a Nursing Home by Given Time Points, Full Sample
eTable 2. Total Number of Nursing Home Residents Who Were Alive and in a Nursing Home by Given Time Points, Limited to Those Initially Non-Medicaid-Enrolled
eFigure 2. Time Until Spend-Down Among Nursing Home Residents Initially Non-Medicaid Enrolled
eFigure 3. Spend-Down Rate Among Nursing Home Residents Initially Non-Medicaid-Enrolled (2015-2019 Cohort), by Length of Stay
eFigure 4. Spend-Down Rate Among Nursing Home Residents Initially Non-Medicaid-Enrolled Before (2018-2019) and During (2020-2021) the Pandemic, by Length of Stay

This supplementary material has been provided by the authors to give readers additional information about their work.

© 2025 Aboulafia G et al. *JAMA Network Open.*

eMethods.

(a) 2018-2022 Nursing Home Resident Cohort Construction
We provide a consort diagram in **eFigure 1** to show how we construct our cohort of nursing home residents who newly entered a nursing home in 2018, by using linked data from the Minimum Dataset (MDS), 2018-2022; Master Beneficiary Summary File (MBSF), 2018-2022; and Medicare Provider Analysis and Review file (MedPAR), 2018-2019. We first start with the 2018 MDS to identify those who newly entered a nursing home in 2018, then append the 2019-2022 MDS datasets to follow the residents for up to five years. We then merge the MDS cohort with the MBSF to obtain monthly Medicaid enrollement status and then merge the MDS-MBSF-linked cohort with the MedPAR to account for Medicare-covered nursing home days.

(b) Additional details on merging MDS-MBSF-linked cohort with the MedPAR file
After obtaining a clean cohort of nursing home residents from the MDS whose coverage status we are able to track through the MBSF, we then linked this cohort to the MedPAR (also described as step 8 in **eFigure 1**), to identify nursing home residents who came in with Medicare covering at least the first part of their stay through the 100-day skilled nursing facility (SNF) benefit. Our process for matching a SNF claim reported in the MedPAR to a nursing home stay reported in the MDS was as follows:

1. We linked a SNF claim to a nursing home stay if the SNF claim occurred within the stay as documented by the MDS (e.g., if a resident had a nursing home stay of May 1, 2018 – December 31, 2018 and a SNF stay of May 2, 2018 – July 30, 2018, the MDS record and MedPAR claim were matched).
2. This led to some MDS and MedPAR records being linked even though their dates didn't exactly align (as in the example above). If the start date of a MedPAR claim was within 14 days of the entry date as recorded by the MDS, we made the assumption that the MedPAR claim was correct and the MDS was misreported, and changed the MDS entry date to reflect the first day of the MedPAR SNF claim (e.g., we would change the MDS record in the above example to an entry date of May 2, 2018 to reflect the MedPAR claim).
    a. If the MDS and MedPAR records were matched but the SNF claim started 14 days after the MDS record started, we made the assumption that the MDS record was correct and the SNF claim was likely for a period of time after the resident had been in the nursing home (as a non-Medicare resident) but then had some kind of temporary hospitalization and returned to the facility with Medicare-covered days. Because these individuals were already observed as Medicaid enrolled or non-Medicaid enrolled at time of admission, we did not account for these hospitalizations.
3. Relatedly, because of misreporting in the MDS, we also identified a subset of individuals (just over 10,000 people) who had SNF claims that extended beyond their nursing home stay as reported by the MDS.
    a. If the MedPAR SNF claim said a resident was covered by Medicare for <=100 days, we corrected the MDS record to reflect the dates of MedPAR claim, and made the assumption that the MDS discharge date was incorrectly reported (e.g., if a resident had a nursing home stay documented by the MDS from May 1, 2018 – June 15, 2018 and the same individual had a SNF claim documented by the MedPAR from May 1 2018 – June 30, 2018, we assumed the MedPAR was correct, and updated the MDS record to reflect the individual was covered by Medicare for the entirety of their stay).
    b. If the MedPAR SNF claim said a resident was covered by Medicare for > 100 days (the minority of this group), we assumed the SNF claim was incorrect and replaced the MedPAR claim end date with the most recent MDS assessment/discharge date.

© 2025 Aboulafia G et al. *JAMA Network Open.*

eFigure 1. CONSORT Diagram of Nursing Home Resident Cohort Data Merging Process

(1) To build our cohort, we start with **2,030,658** individuals in the 2018 MDS who were newly admitted to a nursing home in 2018. To include only those who were newly admitted to a nursing home, we looked back at the 2017 MDS, excluding individuals in the 2018 MDS who were also recorded in the 2017 MDS. We did this to follow a "clean" cohort with no prior nursing home stays from the past year.

(2) Given our interest in measuring time until spend down while an individual is in a facility, we follow people with clean nursing home stays. We defined a "clean stay" as having a gap of 30 days or less between stays (either at the same or different facility). We dropped **712,029** individuals with more than a 30-day gap between stays.

(3) Our cleaned MDS cohort contains **1,318,629** individuals.

(4) We then link this cohort with the MBSF to track coverage status. Because we can only track Medicare beneficiaries in the MBSF, we then drop **107,820** individuals who were in our MDS cohort but not in the MBSF by 2018.

(5) Because we can only track coverage status when an individual is Medicare-eligible, we then drop **16,109** individuals who are in the MBSF but become Medicare-eligible *after* the beginning of our study period (Jan 2018). This number also includes a minority of individuals (n=112) who erroneously have multiple Medicare coverage start dates.

(6) We also drop **123,949** individuals who were a partial dual for at least one month during our study period.

(7) Our cleaned MDS-MBSF-linked cohort contains **1,070,751** individuals.

(8) Then, to determine if an individual was covered by Medicare upon admission to a nursing home, we linked our cohort to MedPAR SNF claims. After data cleaning (described in part (b) of the eMethods), we dropped **9,507** individuals from our MDS-MBSF-linked cohort.

(9) We then drop **394,751** individuals covered by MA for at least one month of our study period, as MedPAR does not reliably capture Medicare-covered days for those with MA.

(9) Finally, we limit our cohort to residents who either remained in a nursing home beyond the Medicare-covered portion of their stay or had no Medicare-covered days, as those with fully Medicare-covered stays cannot be observed starting on or transitioning to Medicaid. We exclude **475,077** individuals with entirely Medicare-covered stays.

(9) Our final MDS-MBSF-MedPAR-linked cohort contains **191,416** individuals.

© 2025 Aboulafia G et al. *JAMA Network Open.*

Notes: Those who we dropped in our cohort construction process are identified on the right side in **red**. MDS = Minimum Dataset; MBSF = Master Beneficiary Summary File; MedPAR = Medicare Provider Analysis and Review; SNF = skilled nursing facility. MA = Medicare Advantage.

© 2025 Aboulafia G et al. *JAMA Network Open.*

eTable 1. Total Number of Nursing Home Residents Who Were Alive and in a Nursing Home by Given Time Points, Full Sample

	**# of residents alive and in a nursing home at given time point**			**# of residents discharged before next given time point**			**# of residents who died before next given time point**		
	Total	*Entered with SNF benefit*	*Entered without SNF benefit*	*Total*	*Entered with SNF benefit*	*Entered without SNF benefit*	*Total*	*Entered with SNF benefit*	*Entered without SNF benefit*
**Full sample**	191,416	90,077	101,339	56,296	19,426	36,870	44,910	21,180	23,730
**3 months**	90,210	49,471	40,739	8,122	3,938	4,184	9,815	5,201	4,614
**6 months**	72,273	40,332	31,941	5,499	3,008	2,491	10,998	5,980	5,018
**1 year**	55,776	31,344	24,432	5,100	3,248	1,852	15,289	8,686	6,603
**2 years**	35,387	19,410	15,977	2,954	1,723	1,231	10,169	5,266	4,903
**3 years**	22,264	12,421	9,843	5,440	4,233	1,207	5,117	2,663	2,454
**4 years**	11,707	5,525	6,182	-	-	-	-	-	-

© 2025 Aboulafia G et al. *JAMA Network Open.*

eTable 2. Total Number of Nursing Home Residents Who Were Alive and in a Nursing Home by Given Time Points, Limited to Those Initially Non-Medicaid-Enrolled

	**# of residents alive and in a nursing home at given time point**			**# of residents discharged before next given time point**			**# of residents who died before next given time point**		
	Total	*Entered with SNF benefit*	*Entered without SNF benefit*	*Total*	*Entered with SNF benefit*	*Entered without SNF benefit*	*Total*	*Entered with SNF benefit*	*Entered without SNF benefit*
**Full sample**	126,626	55,164	71,462	44,056	14,175	29,881	33,957	15,785	18,172
**3 months**	48,613	25,204	23,409	5,089	2,189	2,900	6,144	3,107	3,037
**6 months**	37,380	19,908	17,472	3,058	1,493	1,565	6,547	3,418	3,129
**1 year**	27,775	14,997	12,778	2,302	1,405	897	8,786	4,807	3,979
**2 years**	16,687	8,785	7,902	1,230	682	548	5,608	2,788	2,820
**3 years**	9,849	5,315	4,534	2,562	2,007	555	2,643	1,317	1,326
**4 years**	4,644	1,991	2,653	-	-	-	-	-	-

© 2025 Aboulafia G et al. *JAMA Network Open.*

eFigure 2. Time Until Spend-Down Among Nursing Home Residents Initially Non-Medicaid Enrolled

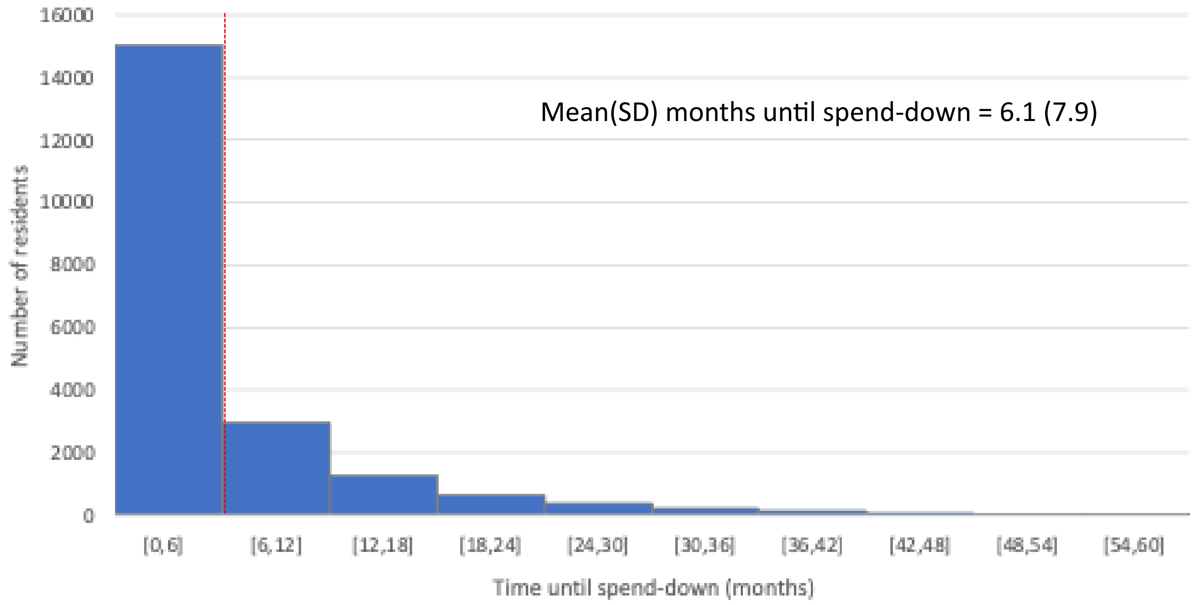

Figure shows the number of 2018 new nursing home entrants who were initially non-Medicaid-enrolled who spent down their assets and became enrolled in Medicaid (N = 20,773) by length of time (in months) until spending down. The red dotted line represents the average time (in months) until spend-down. Those initially non-Medicaid-enrolled includes those non-Medicaid-enrolled upon admission or after the end of their Medicare-covered SNF days, if applicable. Data are from the authors' analysis of the Minimum Dataset (MDS), 2018-2022; Master Beneficiary Summary File (MBSF), 2018-2022; and Medicare Provider Analysis and Review file (MedPAR), 2018-2019.

© 2025 Aboulafia G et al. *JAMA Network Open.*

eFigure 3. Spend-Down Rate Among Nursing Home Residents Initially Non-Medicaid-Enrolled (2015-2019 Cohort), by Length of Stay

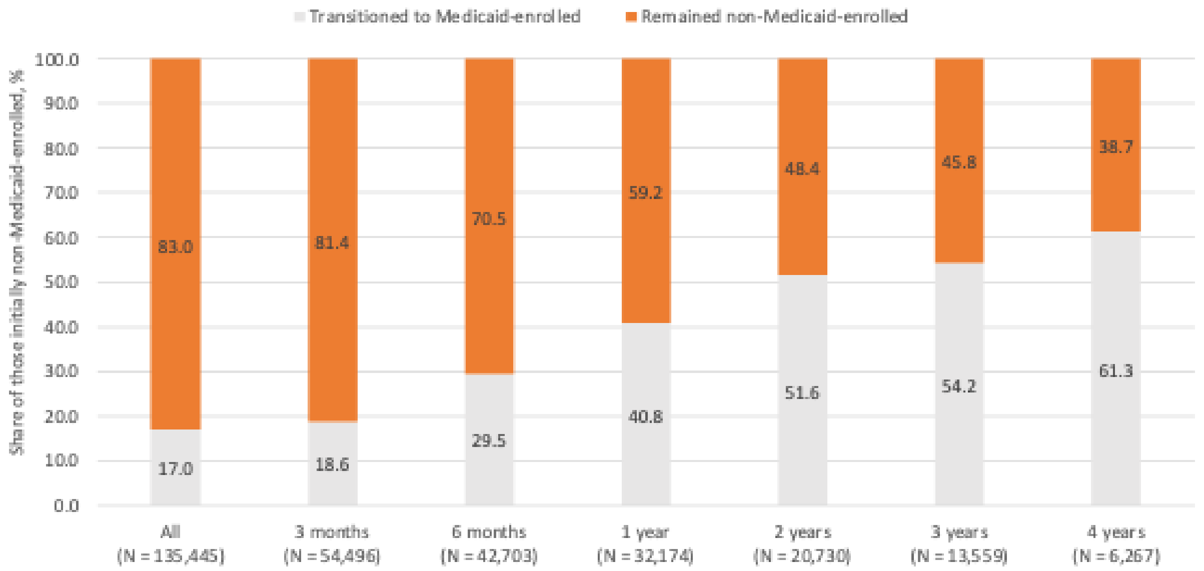

Figure shows the share of 2015 new nursing home entrants who were initially non-Medicaid-enrolled: those non-Medicaid-enrolled upon admission or after the end of their Medicare-covered SNF days, if applicable. "Length of stay" references the number of days an individual remained in a nursing home beyond their Medicare-covered SNF days, if applicable. For individuals admitted directly from the community without any Medicare-covered SNF days, "length of stay" references the true amount of time spent in a nursing home. These individuals were followed for up to five years, between 2015-2019. At each time point, the numerator is the number of individuals alive and in a nursing home who had spent-down by the given time point; the denominator is all individuals alive and in a nursing home who were non-Medicaid-enrolled by that same time point. In the "All" column, the denominator is the full sample who was initially non-Medicaid-enrolled, not conditioning by any time point.The changing denominators are represented by the "N" under each column. Data are from the authors' analysis of the Minimum Dataset (MDS), 2015-2019; Master Beneficiary Summary File (MBSF), 2015-2019; and Medicare Provider Analysis and Review file (MedPAR), 2015-2016.

© 2025 Aboulafia G et al. *JAMA Network Open.*

eFigure 4. Spend-Down Rate Among Nursing Home Residents Initially Non-Medicaid-Enrolled Before (2018-2019) and During (2020-2021) the Pandemic, by Length of Stay

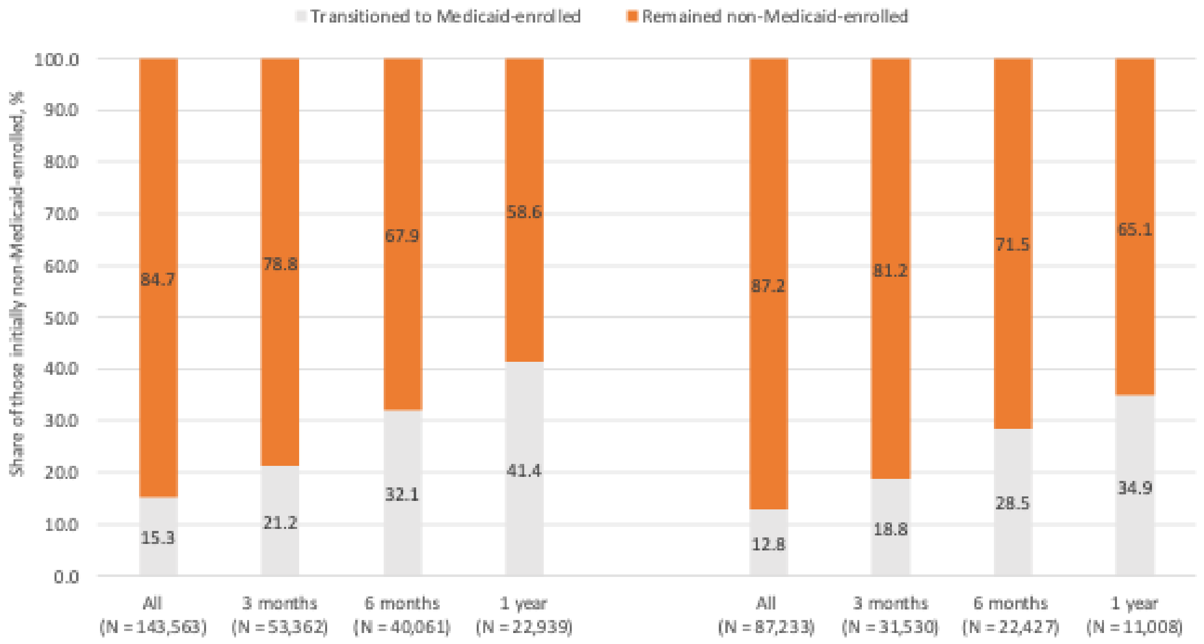

Figure compares the share of 2018 new nursing home entrants ("pre-COVID cohort") who were initially non-Medicaid-enrolled (left side) to the share of 2020 new nursing home entrants ("COVID cohort") who were initially non-Medicaid-enrolled (right side). "Length of stay" references the number of days an individual remained in a nursing home beyond their Medicare-covered SNF days, if applicable. For individuals admitted directly from the community without any Medicare-covered SNF days, "length of stay" references the true amount of time spent in a nursing home. Those initially non-Medicaid-enrolled includes those non-Medicaid-enrolled upon admission or after the end of their Medicare-covered SNF days, if applicable. Both cohorts were followed for up to two years, between 2018-2019 (pre-COVID cohort) and 2020-2021 (COVID cohort). At each time point, the numerator is the number of individuals alive and in a nursing home who had spent-down by the given time point; the denominator is all individuals alive and in a nursing home who were non-Medicaid-enrolled by that same time point. In the "All" column, the denominator is the full sample who was initially non-Medicaid-enrolled, not conditioning by any time point. The changing denominators are represented by the "N" under each column. Data are from the authors' analysis of the Minimum Dataset (MDS), 2018-2019 and 2020-2021; Master Beneficiary Summary File (MBSF), 2018-2019 and 2020-2021; and Medicare Provider Analysis and Review file (MedPAR), 2018 and 2020.

© 2025 Aboulafia G et al. *JAMA Network Open.*
