# Supplementary material for: Asset Spend-Down and Medicaid Enrollment in Nursing Homes
Source: JAMA Netw Open. 2025 Dec 4;8(12):e2546876. doi: 10.1001/jamanetworkopen.2025.46876 (PMC12679321; doi:10.1001/jamanetworkopen.2025.46876)
Supplement: Supplement 2. — Data Sharing Statement [file jamanetwopen-e2546876-s002.pdf]

Data Sharing Statement

Aboulafia. Asset Spend-Down and Medicaid Enrollment in Nursing Homes. *JAMA Netw Open*. Published December 04, 2025. doi:10.1001/jamanetworkopen.2025.46876

Data
Data available: No
